# Gesneriaceae in China and Vietnam: Perfection of taxonomy based on comprehensive morphological and molecular evidence

**DOI:** 10.3897/phytokeys.157.56842

**Published:** 2020-08-26

**Authors:** Wen-Hong Chen, Fang Wen, Ming-Xun Ren, Lihua Yang, Xin Hong, Zhi-Jing Qiu, Yu-Min Shui

**Affiliations:** 1 CAS Key Laboratory for Plant Diversity and Biogeography of East Asia, Kunming Institute of Botany, Chinese Academy of Sciences, 132 Lanhei Road, CN-650201, Kunming, Yunnan Province, China Chinese Academy of Sciences Kunming China; 2 Gesneriad Conservation Center of China (GCCC) & Guangxi Key Laboratory of Plant Conservation and Restoration Ecology in Karst Terrain, Guangxi Institute of Botany, Guangxi Zhuang Autonomous Region and Chinese Academy of Sciences, Guilin, CN-541006, China Guangxi Institute of Botany Guilin China; 3 Center for Terrestrial Biodiversity of the South China Sea, College of Ecology and Environment, Hainan University, Haikou, CN-570228, China Hainan University Haikou China; 4 Key Laboratory of Plant Resources Conservation and Sustainable Utilization, South China Botanical Garden, Chinese Academy of Sciences, Guangzhou, CN-510650, China Chinese Academy of Sciences Guangzhou China; 5 Anhui Provincial Engineering Laboratory of Wetland Ecosystem Protection and Restoration, School of Resources and Environmental Engineering, Anhui University, CN-230601, Hefei City, Anhui Province, China Anhui University Hefei China; 6 Key Laboratory of Southern Subtropical Plant Diversity, Fairy Lake Botanical Garden, Shenzhen and Chinese Academy of Sciences, Shenzhen, CN-518004, China Shenzhen and Chinese Academy of Sciences Shenzhen China

## Abstract

No

Morphology is fundamental to taxonomy. Specimens in herbaria can provide unique supporting bases for scientific nomenclature. However, they usually reveal some limited variation of the taxa in nature and need to be revised gradually in future taxonomic studies. Because botanists make taxonomic treatments in herbaria without the benefit of molecular verification, many synonyms can occur. Traditionally, morphological treatment needs a combination of detailed herbaria work and extensive fieldwork. In general, the former work is usually dull, requires considerable patience, and tends to be neglected; this leads to unsubstantiated new synonyms. On the converse, field observations benefit from high-tech tools and equipment, which can reveal more delicate and detailed content in the field and the laboratory. These include detailed images directly from field observation by digital cameras, micro-morphology from SEM, and Vertical microscope work. In a word, the absence of detailed morphology from herbaria and the field cannot support good taxonomic work.

Diligent molecular work can support taxonomic revision. At the species level, molecular phylogeny seldom provides direct evidence to confirm a new species, but only tells us its affinities logically (Chen et al. 2014). Molecular evidence is not usually considered when new species are described. Although morphology seems to work in Gesneriaceae at the genus level, exceptions in morphology often happen, particularly with some of the expanded genera in Asia (*Oreocharis*, *Petrocodon*, and *Primulina*) (Figure 1; Möller et al. 2011; Wang et al. 2011; Weber et al. 2011a, b). In such cases, molecular evidence is helpful for accurate taxonomic treatment. However, the next crucial question will be how many DNA sequences will support the well-resolved relationships of the taxa above the species level. Based on the recent study, it seems to be that the combination of ITS and *trn*L-F is not enough to resolve the relationship within the above expanded genera. In special cases, we strongly suggest adopting more sequences to issue the taxonomic revision in the future study of Gesneriaceae (Chen et al. 2020), such as *atp*B-*rbc*L, *ndh*H-*rps*15-*ycf*1, *rpl*132, *trn*C-*trn*D, *trn*L-F, *trn*T-trnL of chloroplast DNA.

**Figure 1.**
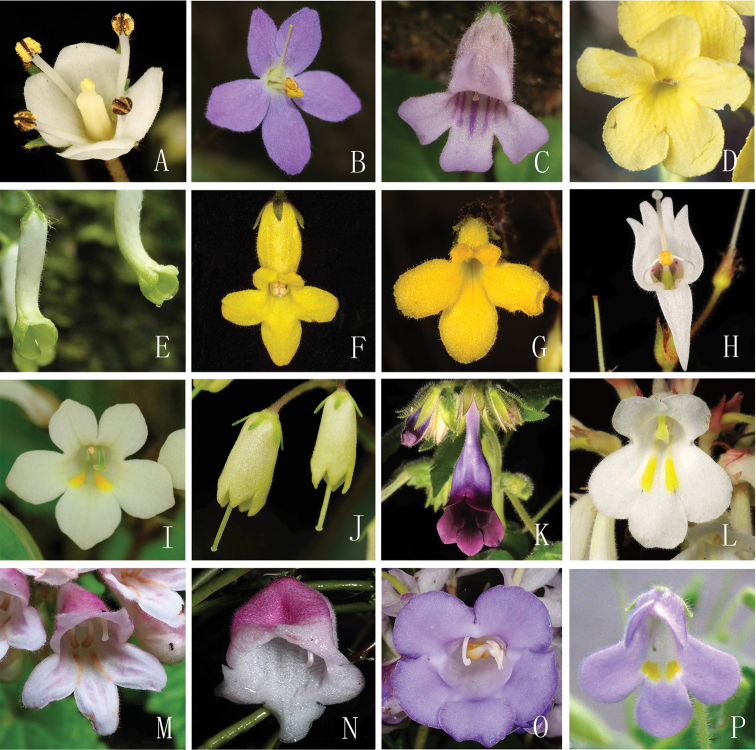
Flowers of some species of Gesneriaceae in China and Vietnam **A***Bournea sinensis* Oliv. (photographed by Yu-Min Shui) **B***Oreocharis guileana* (B.L. Burtt) Li H. Yang & F. Wen, comb. nov. (by Li-Hua Yang) **C***Oreocharis baolianis* (Q.W. Lin) Li H. Yang & M. Kang, comb. nov. (by Li-Hua Yang) **D***Oreocharis jasminina* S.J.Ling, F.Wen & M.X. Ren, sp. nov. (by Shao-Jun Ling) **E***Oreocharis flavovirens* Xin Hong (by Xin Hong) **F***Oreocharis wumengensis* Lei Cai & Z.L.Dao, sp. nov. (by Lei Cai) **G***Oreocharis fulva* W.H.Chen & Y.M.Shui, sp. nov. (by Yu-Min Shui) **H***Allocheilos rubroglandulosus* W.H. Chen & Y.M. Shui, sp. nov. (by Yu-Min Shui) **I***Petrocodon rubiginosus* Y.G.Wei & R.L.Zhang, sp. nov. (by Fang Wen) **J***Petrocodon luteoflorus* Lei Cai & F. Wen, sp. nov. (by Fang Wen) **K***Deinostigma fasciculatum* W.H.Chen & Y.M.Shui, sp. nov. (by Yu-Min Shui) **L***Primulina xuansonensis* W.H.Chen & Y.M.Shui, sp. nov. (by Yu-Min Shui) **M***Didymocarpus lobulatus* F. Wen, Xin Hong &W.Y. Xie, sp. nov. (by Jia-Jun Zhou) **N***Paraboea myriantha* Y.M. Shui & W.H. Chen, sp. nov. (by Yu-Min Shui) **O**Paraboea sinensis var. glabrissima W.H.Chen & Y.M.Shui, var. nov. (by Yu-Min Shui) **P***Petrocosmea nanchuanensis* Z.Y. Liu, Z.Y. Li & Z.J. Qiu, sp. nov. (by Zhi-Jing Qiu).

Some detailed rules are suggested during taxonomic revision in Gesneriaceae. First, the new species’ establishment is usually based on morphological differences, with at least two or more different characteristics in diagnosis. It would be better to provide the key to the new species suggested and their related groups and species. Second, the comprehensive observation of morphology is necessary to support the new species, such as staminodes, discs of flowers, and the abaxial surface of leaves. Third, statistical analysis of morphological characters using sufficient samples from multiple populations can provide unbiased evidences for the taxonomic treatment of some species with subtle morphological differences (e.g. Yang et al. 2019). Fourth, chromosomes and pollen grains are important to the taxonomic revision and are strongly encouraged (Pan 1987; Yang et al. 2020). Lastly, more DNA sequences such as *atp*B-*rbc*L, *ndh*H-*rps*15-*ycf*1, *rpl*132, *trn*C-*trn*D, *trn*L-F, *trn*T-trnL, *psb*A-*trn*H than should be considered during the taxonomic treatment together with ITS (Qiu et al. 2015; Roalson and Roberts 2016; Chen et al. 2020).

This special issue focuses on China and Vietnam: an essential center of biodiversity worldwide (Myers et al. 2000). Gesneriaceae includes more than 700 accepted species in the area, and thus provides a suitable example for answering the above taxonomic questions (Ho 2000; Myers et al. 2000; Wen et al. 2019). Tan et al. (2020) offer an in-depth look at the updated taxonomy and biogeographical patterns of Asian Gesneriaceae. Hainan Island, one of the biggest islands in China and Vietnam, harbours an extremely high endemism ratio of Gesneriaceae and all *Oreocharis* species on this island are endemic (Ling et al. 2017). With an extensive examination combining both morphological and molecular evidences, Ling et al. (2020a, b) explored the taxonomical treatment of Hainan *Oreocharis* and found a possible new species. In addition to the numerous new species’ taxonomic treatments referred to, several studies in this special issue emphasize the use of comprehensive morphological observation and more molecular data to provide convincing conclusions. It would be desirable that all discoveries and taxonomic revisions will be conducted under these strict criteria suggested here in the future.
